# Exposure to Low-Dose Bisphenol A Impairs Meiosis in the Rat Seminiferous Tubule Culture Model: A Physiotoxicogenomic Approach

**DOI:** 10.1371/journal.pone.0106245

**Published:** 2014-09-02

**Authors:** Sazan Ali, Gérard Steinmetz, Guillaume Montillet, Marie-Hélène Perrard, Anderson Loundou, Philippe Durand, Marie-Roberte Guichaoua, Odette Prat

**Affiliations:** 1 Institut Méditerranéen de Biodiversité et d'Ecologie marine et continentale (IMBE), Centre National de la Recherche Scientifique (CNRS) UMR 7263/ Institut de Recherche pour le Développement (IRD) 237, Faculté de Médecine, Aix-Marseille Université (AMU), Marseille, France; 2 Institute of Environmental Biology and Biotechnology (IBEB), Life Science division, French Alternative Energy and Atomic Energy Commission (CEA), Marcoule, Bagnols-sur-Cèze, France; 3 Institut de Génomique Fonctionnelle de Lyon (IGFL), Centre National de la Recherche Scientifique (CNRS) UMR 5242/ Institut National de la Recherche Agronomique (INRA), Ecole Normale Supérieure de Lyon (ENS), Lyon, France; 4 Unité d'Aide Méthodologique à la Recherche clinique, Faculté de Médecine, Aix-Marseille Université (AMU), Marseille, France; Nanjing Medical University, China

## Abstract

**Background:**

Bisphenol A (BPA) is one of the most widespread chemicals in the world and is suspected of being responsible for male reproductive impairments. Nevertheless, its molecular mode of action on spermatogenesis is unclear. This work combines physiology and toxicogenomics to identify mechanisms by which BPA affects the timing of meiosis and induces germ-cell abnormalities.

**Methods:**

We used a rat seminiferous tubule culture model mimicking the *in vivo* adult rat situation. BPA (1 nM and 10 nM) was added to the culture medium. Transcriptomic and meiotic studies were performed on the same cultures at the same exposure times (days 8, 14, and 21). Transcriptomics was performed using pangenomic rat microarrays. Immunocytochemistry was conducted with an anti-SCP3 antibody.

**Results:**

The gene expression analysis showed that the total number of differentially expressed transcripts was time but not dose dependent. We focused on 120 genes directly involved in the first meiotic prophase, sustaining immunocytochemistry. Sixty-two genes were directly involved in pairing and recombination, some of them with high fold changes. Immunocytochemistry indicated alteration of meiotic progression in the presence of BPA, with increased leptotene and decreased diplotene spermatocyte percentages and partial meiotic arrest at the pachytene checkpoint. Morphological abnormalities were observed at all stages of the meiotic prophase. The prevalent abnormalities were total asynapsis and apoptosis. Transcriptomic analysis sustained immunocytological observations.

**Conclusion:**

We showed that low doses of BPA alter numerous genes expression, especially those involved in the reproductive system, and severely impair crucial events of the meiotic prophase leading to partial arrest of meiosis in rat seminiferous tubule cultures.

## Introduction

Bisphenol A (4, 4′-isopropylidenediphenol), or BPA, is one of the world's most highly produced chemicals, used to manufacture epoxy resins and polycarbonate plastics. According to physiologically based pharmacokinetic studies, BPA is found in human serum, urine, milk and fat, with plasma levels ranging from 0.2 to 20 ng/mL (or 1 to 100 nM) [1], [2], [3], [4], [5], [6]. This substance is mainly absorbed by the digestive tract. BPA is an endocrine-disrupting chemical (EDC), which could interact with both α- and β-estrogen receptors [7] and bind to androgen receptors [8]. Emerging evidence suggests that this molecule may influence multiple endocrine-related pathways [9]. Several controversies have divided scientific opinion regarding the adverse effects of BPA in the testis and reproductive organs [10], [11]. Indeed, previous studies indicate that there are no reproductive effects of BPA [12], [13], [14], [15]. Nevertheless, numerous findings suggest that BPA adversely affects the male reproductive system. Tohei et al [16] showed that bisphenol A inhibits testicular function in adult male rats. In mice, testicular hypotrophy and decreased daily sperm production were observed in the presence of BPA [17], [18]. In humans, BPA also reduced sperm concentration, motility and morphology [19]. BPA exposure may also induce apoptosis in rat germ cells *in vivo* [20] and in cultured rat Sertoli cells [21], and has the potential to redistribute several known Sertoli cell junctional proteins [22], [23]. Subsequent studies also demonstrated that BPA is genotoxic. The accumulation of DNA damage in germ cells was induced by BPA exposure via oxidative stress [24]. BPA causes meiotic abnormalities in oocytes [25], [26], [27], [28], [29], [30] and in male germ cells of the adult rat [31]. Despite these numerous studies of the effects of BPA on sperm quality, few investigations have been conducted on the crucial meiotic step of spermatogenesis [24], [32]. Thus, the molecular action of BPA on spermatogenesis remains largely unknown.

We conducted a fine analysis of the first meiotic prophase with low doses of BPA (1 nM and 10 nM), approximating levels in biological fluids [33]. Decreased efficiency of sperm production in mice appeared at a dose of 20 µg/kg/day (20 ng/g body weight/day) [34]. This study was performed using a validated rat seminiferous tubule culture model [35], able to reproduce spermatogenesis *ex vivo*. This model allows the analysis of cellular responses induced by exposure to low doses of toxic substances for three weeks. This period of time corresponds, in the rat, to the development of spermatogenesis and mimics puberty, a critical period of life with regard to endocrine disruptors [36], [37].

It appears that a model “sensitive” to the possible adverse effects of chemicals is indeed of the highest importance for toxicological studies. These models must be able to respond to very low concentrations of toxicants. It must also be underlined that, in order to prevent “false-positive” results, particular attention must be paid to toxicant test concentrations, which must be realistic. Our intention was to investigate whether BPA, at the selected doses for three weeks, could alter the chronology of meiosis and induce morphological abnormalities, and to apprehend its mechanisms of action, combining toxicogenomic and physiological approaches.

Microarray-based transcriptional profiling is a powerful and ultrasensitive tool for monitoring altered cellular functions and pathways under the action of toxicants, providing a wealth of information for sketching the mode of action of toxic substances [38], [39], [40] or for finding new toxicity bioindicators [41]. However, to date very few transcriptome analyses have been conducted to comprehend the molecular action of BPA on spermatogenesis [42], [43], [44]. Using transcriptomics, we were able to detect changes in gene expression at biological doses, in the nanomolar range. We studied whether specific patterns of gene modulation could be associated with cytological changes of the first meiotic prophase, observed by immunostaining of the synaptonemal complexes (SC) with an anti-SCP3 antibody. This model allowed for immunocytochemistry and transcriptomic experiments using the same cultures at the same exposure times.

## Material and Methods

### Animals

The entire study was performed *ex vivo* using cultures of seminiferous tubules. For these cultures, Male 23-day-old Sprague Dawley rats from Charles River France Inc. (supplier: Janvier, France), having undergone no treatment, were used. Animals were housed 3/ cage, at temperature 21±3°C, light cycle 12–12 (6 pm-6 am), diet made of SDS VRF1(from Special Diets Services), water filtered 0.1 µm in bottle system, sawdust bedding, autoclaved. Rats were anesthetized with chloroform then decapitated. At the age of 22–23 days the most advanced germ cells are late spermatocytes [45] allowing to study the whole meiotic phase under our culture conditions [35].

In order to counterbalance interanimal variations, testes from eight rats were pooled in every culture, and used immediately, as previously described [35].

The same population was seeded for control cultures and cultures exposed to toxicant. Analyses were performed at days 8 (D8), 14 (D14) and 21 (D21) of the cultures. All procedures were approved by the Scientific Research Agency (approval number 69306) and conducted in accordance with the guidelines for care and use of laboratory animals. The experimental protocol was designed in compliance with recommendations of the European Economic Community (EEC) (86/ 609/EEC) for the care and use of laboratory animals.

### Preparation and culture of seminiferous tubules

The technique of seminiferous tubule culture has been described previously [35]. Cultures were performed with and without BPA. When required, BPA was added beginning from day 2, at 1 nM or 10 nM (Sigma-Aldrich Corporation, St. Louis, USA) in the basal compartment of the bicameral culture chamber. BPA concentrations were selected on the basis of those found in human and rat plasma, i.e. 0.2 to 20 ng/mL, meaning 1 to 100 nM [1], [2], [3], [4], [6]. 0.3% DMSO was used as the BPA dilution vehicle; the same solvent concentration was introduced in control cultures.

### RNA extraction, labelling and microarray experiments

Two different pools of seminiferous tubules were exposed to two concentrations of BPA (1 nM and 10 nM) or to complete medium with vehicle (control cells) for 8, 14, and 21 days. Total RNA was extracted using the RNeasy Mini kit (Qiagen). RNAs were quantified with the Nanodrop 1000 spectrophotometer; their qualities were assessed with the Agilent 2100 Bioanalyzer. RNA samples were amplified and labeled with the cyanine-3 fluorophore using a Low Input QuickAmp Labeling Kit (Agilent). Hybridization was performed using Agilent Oligo Microarrays (Rat V3 4×44K). Fluorescence was scanned and signal data were extracted with Feature Extraction Software (Agilent).

### Cytological methods

#### Samples treatment

Spreading, and immunocytological localization of SC axial and lateral elements, were performed according to [46]. After spreading by cytocentrifugation at 30 g, slides were fixed in 2% paraformaldehyde (Merk Darmstad, Germany). A rabbit polyclonal anti-SCP3 antibody (Abcam, Cambridge, UK Ab 15093) was used at a 1∶100 dilution, to reveal axial elements and lateral elements of the SC. Detection was performed with an FITC-conjugated anti-goat immunoglobulin G (Abcam, Cambridge, UK) at a dilution of 1∶100. Slides were mounted in antifade medium (Vectashield, Vector Laboratories, Burlingame, USA).

#### Microscope analysis

A Zeiss Axioplan 2 Fluorescence Photomicroscope (Carl Zeiss, Oberkochen, Germany) was used to observe the spermatocyte nuclei. Primary spermatocytes, stained with the anti-SCP3 antibody, were selected to evaluate the respective percentages of leptotene, zygotene, pachytene and diplotene stages: 100 to 200 nuclei were analyzed for each culture, for control cultures, and for each time and BPA dose condition. We evaluated the percentages of the three pachytene substages, P1, P2 and P3, corresponding to early, mid and late pachytene substages, in the rat [37]. These substages were defined according to the condensation degree of the sex bivalent during the pachytene stage; 50 nuclei were analyzed for each condition. The pachytene index (PI) was evaluated for each culture and for each time and BPA dose. We defined the PI in rat by the ratio P3/P1+P2+P3 [37]. The percentages of nuclei showing SC abnormalities were quantified at each time point, in both control cultures and cultures exposed to 1 nM and 10 nM BPA. For each stage, and for each abnormality, we researched a possible dose-and-time variation.

### Statistical analysis

#### Transcriptomic analysis

In this experimental design, six independent analyses were conducted versus each specific control for the considered time point: a) 1 nM BPA-exposed cells for 8 days, b) 1 nM BPA-exposed cells for 14 days, c) 1 nM BPA-exposed cells for 21 days, d) 10 nM BPA-exposed cells for 8 days, e) 10 nM BPA-exposed cells for 14 days, f) 10 nM BPA-exposed cells for 21 days. For each analysis, eight raw fluorescence data files (four controls and four tests) were submitted to GeneSpring Software GX11 (Agilent Technologies) using a widely used method for determining the significance change of gene expression [38], [47]. The fold change cutoff between control and exposed samples was set to 1.5. Genes significantly up- or downregulated were determined by an unpaired t-test, with a p-value <0.05 and a Benjamini-Hochberg false discovery rate correction. We thus obtained probe sets that were significantly induced or repressed after exposure to BPA.

#### Immunocytochemistry (ICC)

Statistical analysis was performed using PASW Statistics Version 17.0.2 (IBM SPSS Inc., Chicago, IL, USA). Continuous variables are expressed as means±SD. Comparisons of means between two groups were performed using a Student's t-test. All tests were two-sided. The statistical significance was defined as p<0.05. Three biological replicates were analyzed for D8 and D14, and two replicates for D21. Each experiment included controls (vehicle only) and tests (BPA). The total number of nuclei analyzed was 4630, combining all doses and time points.

### Biological analysis

Lists of genes significantly induced or repressed after exposure to BPA were uploaded into Ingenuity Pathway Analysis Software (IPA, Ingenuity Systems, www.ingenuity.com) for biological analysis by comparison with the Ingenuity Knowledge Database. These lists of altered genes were then processed to investigate the functional distribution of these genes, as defined by Gene Ontology. Datasets and known canonical pathways associations were measured by IPA by using a ratio (R) of the number of genes from a dataset that map to a specific pathway divided by the total number of genes that map to this canonical pathway. A Fisher's exact test was used to determine a p-value representing the significance of these associations.

### Quantitative RT-PCR

Total RNA was isolated according to the manufacturer's instructions using the RNeasy Kit (Qiagen), and treated with DNase. RNA purity and concentration were determined by UV on a Nanodrop Spectrophotometer and integrity was assessed on an Agilent 2100 Bioanalyzer (Agilent Technologies). All the samples used in this study showed 28S/18S ratio signing intact and pure RNA. Differential analysis of RNA from cells exposed to NPs and from unexposed cells was performed by qRT-PCR with the Sybr Green PCR Master Mix (Finzyme) Kit according to the manufacturer's instructions, on Opticon II (Biorad). Primer (Sigma) sequences were, for *Stra8*: 5′ CAGCCTCAAAGTGGCAGGTA 3′ (forward) and 5′ GGGAGAGGAGTGGGACAGAT 3′ (reverse); for *Mlh1*: 5′ CGCCATGCTGGCCTTAGATA 3′ (forward) and 5′ CCTCCAAAGGCGGCACATA 3′ (reverse); for *Prdm9*: 5′ AGAATGAGAAAGCCAACAGCA 3′ (forward) and 5′ AGACTCCTTAGAAGTTTTAGCAGA 3′ (reverse); for *Sycp1*: 5′ GAGAGAAGACCGTTGGGCA 3′ (forward) and 5′ TCCATTGCAAGTAAAAGCAACA 3′ (reverse); for *Fpr3*: 5′ ACTGTGAGCCTGGCTAGGAA 3′ (forward) and 5′ CTCGTGAAGCACGGCTAGAA 3′ (reverse); for *Dmc1*: 5′ CTTTCCGTCCAGATCGCCTT 3′ (forward) and 5′ AAAATGCCGGCTTCTTCGTG 3′ (reverse); for *Card11*: 5′ CTCAGGCCCAGTTTCTCCAG 3′ (forward) and 5′ CTGTTGAGCTCTGTGGAGGG 3′ (reverse); for *Nfkb1*: 5′ GGAGATGGCCCACTGCTATC 3′ (forward) and 5′ TTCGGAAGGCCTCGAATGAC 3′ (reverse). For *Stra8, Mlh1, Prdm9, Sycp1, Fpr3, Dmc1, Card11, Nfkb1*, the amplicon sizes were *347, 200, 100, 286, 313, 160, 92, and 223* bp, respectively. The measurements were the means of six individual results and normalization was based on the total RNA mass quantified on the Nanodrop. Expression ratios were calculated according to Pfaffl et al. [48], where the relative expression ratio (R) of a target gene was calculated using PCR efficiency (E) and the CT (number of cycles at threshold) deviation of an unknown sample versus a control. The target gene fold change was then expressed as follows: E ^(CT mean control _ CT mean treatment)^. Statistical significance was tested by Pair Wise Fixed Reallocation Randomization Test (REST software) where a p-value less than 0.05 was considered significant.

## Results

### Transcriptome analysis

**Figure 1:**
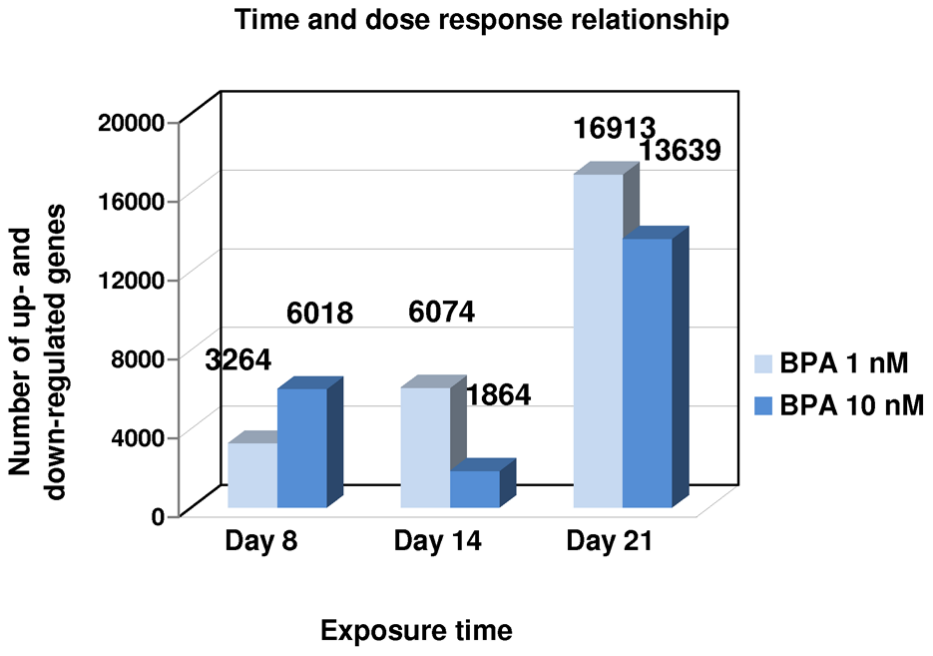
Total number of genes differentially up- or downregulated by 1 nM and 10 nM BPA after 8, 14 and 21 days of exposure. Genes were selected with a fold change cutoff ≥1.5 (p-value <0.05). The global expression change compared with control cells was time but not dose dependent.

Figure 1 indicates the number of significantly differentially expressed genes for each dose and time point. These figures encompass up- and downregulated transcripts. The number of genes affected by BPA increased markedly over the exposure time. At 1 nM BPA, this modification was time dependent. At 10 nM, there was, curiously, a decrease in the number of modulated genes at D14, but this number increased again at D 21. The entire list of significantly up- and downregulated genes for each dose and time point (FC>1.5 with p-value <0.05) is provided as supplementary material (Table S1).

#### Altered physiological functions and canonical pathways

**Figure 2:**
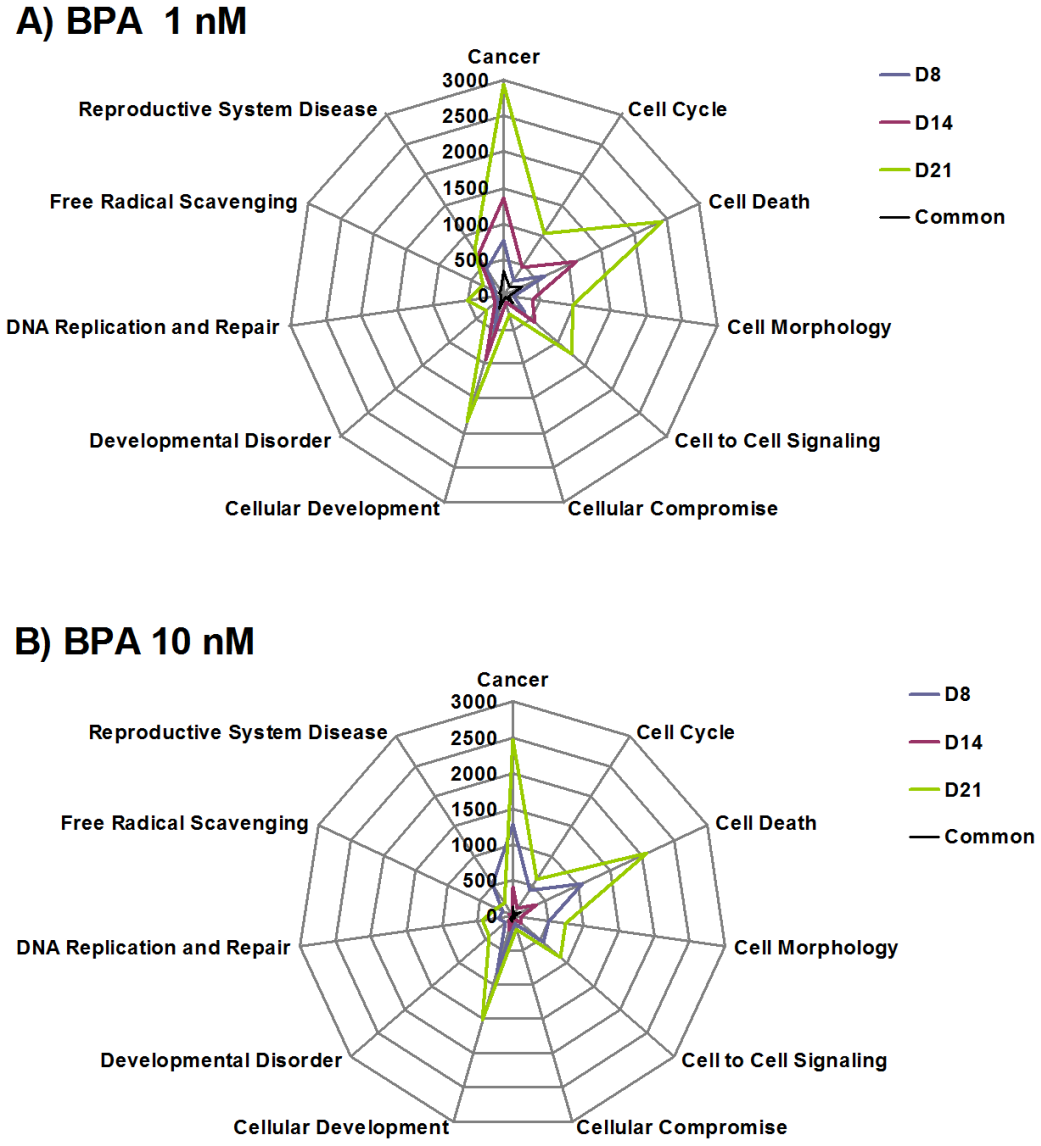
Distribution of differentially expressed genes per altered function. Genes significantly up- or downregulated after cell exposure to BPA for 8 days (blue), 14 days (red), or 21 days (green), and genes common to all time points (black) were examined and classified per function according to Gene Ontology. The results comprise a specific pattern of BPA toxicity, similar for all time points. The amplitude of the toxicity is given by the scale representing the number of modulated genes for each type of altered function. **A** – 1 nM BPA. **B** – 10 nM BPA.

We analyzed the distribution of altered genes per function, as defined in Gene Ontology (Figure 2), using Ingenuity Pathway Analysis. Radar plots helped to apprehend the complexity of toxicity, both in terms of amplitude and effect. This resulted in a specific pattern representing the toxicity for each individual dose and time point. Graphic overlay of all time points allows a visual comparison of the extension of adverse effects throughout the exposure time. The distribution of functions altered by BPA on the radar plot delineated similar patterns from D8 to D21, meaning that adverse effects were amplified with time but not with dose (Fig. 2A and 2B for 1 nM and 10 nM, respectively). Whatever the dose and time point the top three altered functions were cancer, cell death and cellular development, as shown in Fig. 2. For instance, at D21/1 nM BPA, the numbers of genes related to cancer, cell death and cellular development were 2927, 2422 and 1833, respectively. For reproductive system disease and DNA replication and repair, the numbers of genes were 746 and 511, respectively. For other dose and time points, the numbers of genes per altered function are indicated in Figure 2.

**Figure 3:**
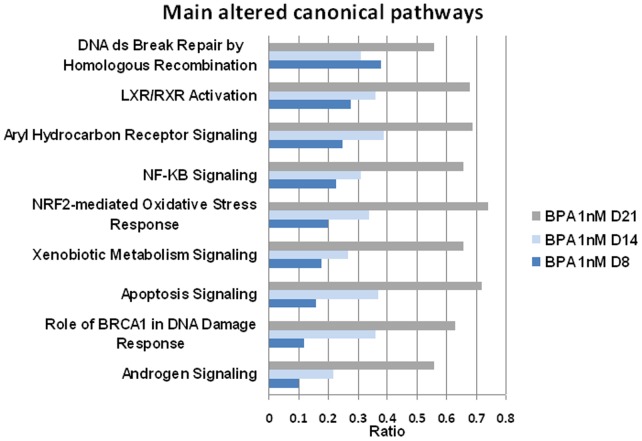
Comparative analysis of canonical pathways significantly altered by 1 nM BPA at D8, D14 and D21. The x-axis depicts gene ratios within a dataset mapping to the considered pathway (see Methods for calculation). A Fisher's exact test was used to determine a p-value representing the significance of these associations (p<0.01). In all cases, the ratios increased with exposure time.

Figure 3 shows the common canonical pathways disturbed by BPA (1 nM at D8, D14, and D21). Each canonical pathway is constituted of a finite number of genes. For each time point, we calculated a ratio indicating the percentage of altered genes in our dataset belonging to a given canonical pathway (for precise calculation, see Material and Methods). We selected nine canonical pathways on the basis of the most significant p-values. These nine main canonical pathways were altered by BPA in a time-dependent manner. The following scores are given for D21/1 nM BPA, but all were altered early, at D8.

*DNA double-strand break repair by homologous recombination,* R = 0.56, p-value 3.52×10^−3^
*LXR/RXR activation, *R = 0.68, p-value 4.26×10^−3^
*Aryl hydrocarbon receptor signaling,* R = 0.69, p-value 2.75×10^−4^
*NF-κB signaling,* R = 0.66, p-value 4.78×10^−3^
*NRF2-mediated oxidative stress response,* R = 0.74, p-value 1.29×10^−8^
*Xenobiotic metabolism signaling,* R = 0.66, p-value 6.76×10^−4^
*Apoptosis signaling, *R = 0.72, p-value 2.95×10^−4^
*Role of BRCA1 in DNA damage response,* R = 0.63, p-value 9.77×10^−3^
*Androgen signaling,* R = 0.56, p-value 2.59×10^−2^

#### Transcription changes of genes expressed in meiotic and premeiotic cells

**Figure 4:**
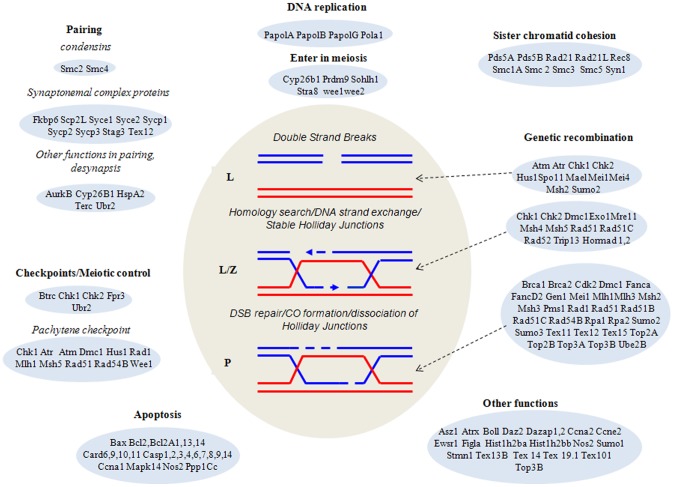
Diagram of the main stages of meiotic recombination and the corresponding stages of the meiotic prophase (L =  leptotene, Z =  zygotene and P =  pachytene). The 120 genes showing a fold change ≥1.5 (p value ≤0.05) in BPA-treated cultures compared with controls, and involved in events of the meiotic prophase, were classified according to their function. This figure shows that the main functions of the first meiotic prophase are altered by BPA. Genes having several functions appear several times in this figure.

**Figure 5:**
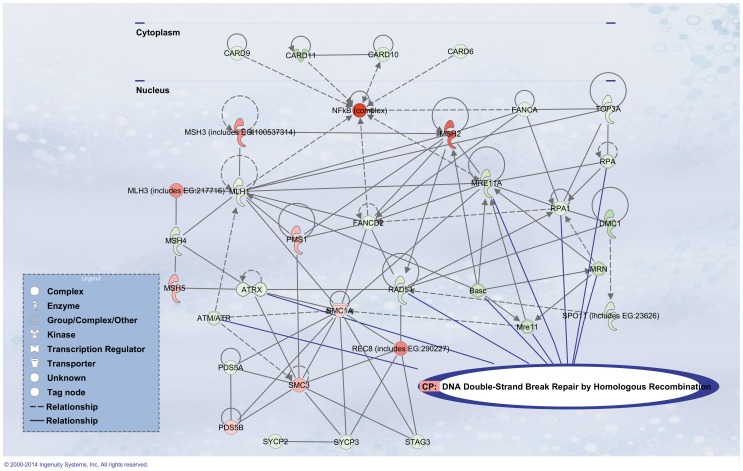
The top-ranked Ingenuity network identified within the group of 120 genes preceding meiotic divisions, involved in the first meiotic prophase, or essential to meiosis that were found to be differentially expressed in our datasets. This literature-based network shows the high level of connections between genes linked to DNA DSB repair in our datasets, considering all BPA doses and time points. The indicated values of fold change (induction or repression) are the maximal values found in the time course with 1 nM and 10 nM BPA. The red nodes are upregulated, the green nodes are downregulated.

Of the 746 deregulated genes of the reproductive system, we focused on 120 genes known to be involved in premeiotic steps or in the first meiotic prophase (Table S2). As for the total set of genes (Fig. 1), the number of BPA-affected genes increased markedly over the time of exposure, except for a decrease at D14/10 nM BPA. The highest fold changes were observed at D21/1 nM. Among these 120 genes, the number of downregulated genes (62.2%) widely exceeded the number of upregulated genes. The genes that had the greatest fold change were downregulated, except for Nos2, which was upregulated. The greatest fold change was observed for Stra8 (−37.83) which was deregulated in all conditions. Figure 4 shows that BPA deregulates genes involved in all the important processes of premeiotic steps and first meiotic prophase. The genes affected with the greatest fold change were mainly involved in meiotic initiation and recombination. Indeed, of the 120 genes, 62 were directly involved in these two functions. Some modified genes were involved in functions other than meiotic events but nevertheless essential to meiosis, such as transcriptional regulation, cell cycle, chromatin organization, protein stability, stress-induced responses and repression of retrotransposable elements. Most of this study's meiotic genes coded for nuclear proteins, some for cytoplasmic proteins, and rarely for plasma membrane proteins (Table S2). All of these genes are represented in Fig. 4. They are classified according to their respective functions in meiotic initiation, recombination and pairing. All have been shown to be interconnected in a network (Fig. 5) obtained by IPA.

#### qRT-PCR validation of the microarray data focused on meiosis

Quantitative RT-PCR was performed using the same batches of RNA as those evaluated by microarrays. Validation of the microarray data was investigated on five genes belonging to BPA-1 nM/D21, and on 7 genes belonging to BPA-10 nM/D21. Table 1 reports the compared fold changes obtained with microarray and qRT-PCR (p-value <0.05).

**Table 1:** Microarray gene expression validation by qRT-PCR.

1 nM BPA, D21			
Gene ID	Primers (5′-3′)	Microarray	qRT-PCR
		fold change	fold change
		BPA/Ctrl	BPA/Ctrl
*Stra8*	F: CAGCCTCAAAGTGGCAGGTA	−37.8	−6.1
	R: GGGAGAGGAGTGGGACAGAT		
*Mlh1*	F: CGCCATGCTGGCCTTAGATA	−3.1	−1.1
	R: CCTCCAAAGGCGGCACATA		
*Prdm9*	F: AGAATGAGAAAGCCAACAGCA	−25.4	−5.6
	R: AGACTCCTTAGAAGTTTTAGCAGA		
*Sycp1*	F: GAGAGAAGACCGTTGGGCA	−9.1	−3.9
	R: TCCATTGCAAGTAAAAGCAACA		
*Fpr3*	F: ACTGTGAGCCTGGCTAGGAA	−25.1	−3.5
	R: CTCGTGAAGCACGGCTAGAA		

10 nM BPA, D21			
Gene ID	Primers (5′-3′)	Microarray	qRT-PCR
		fold-change	fold change
		BPA/Ctrl	BPA/Ctrl
*Stra8*	F: CAGCCTCAAAGTGGCAGGTA	−4.6	−6.9
	R: GGGAGAGGAGTGGGACAGAT		
*Mlh1*	F: CGCCATGCTGGCCTTAGATA	−2.3	−1.6
	R: CCTCCAAAGGCGGCACATA		
*Prdm9*	F: AGAATGAGAAAGCCAACAGCA	−3.9	−2.2
	R: AGACTCCTTAGAAGTTTTAGCAGA		
*Frp3*	F: ACTGTGAGCCTGGCTAGGAA	−2.6	−2.7
	R: CTCGTGAAGCACGGCTAGAA		
*Dmc1*	F: CTTTCCGTCCAGATCGCCTT	3.9	2.5
	R: AAAATGCCGGCTTCTTCGTG		
*Card11*	F: CTCAGGCCCAGTTTCTCCAG	−3.9	−11
	R: CTGTTGAGCTCTGTGGAGGG		
*Nfkb1*	F: GGAGATGGCCCACTGCTATC	3	5.9
	R: TTCGGAAGGCCTCGAATGAC		

Changes in the mRNA expression measured by transcriptomics and by quantitative real-time PCR. The fold changes in cells treated with BPA at 1 nM and 10 nM are expressed versus untreated cells, at D21. For qRT-PCR, the measurements were the means of six measurements (triplicates of two independent experiments) and normalization was based on the total RNA mass quantified on Nanodrop spectrophotometer. All expression levels in treated cells were significantly different from controls (p<0.05). F =  forward; R =  reverse.

### Immunocytological analysis of the meiotic prophase

#### Effect of BPA on the percentage of SCP3-stained meiotic stages

Each stage's normal morphological aspect of the first meiotic prophase – leptotene, zygotene, pachytene and diplotene – has been described previously [37]. The percentages of these four stages in the control cultures were of the same order of magnitude as those obtained previously. These percentages were evaluated from 100 to 200 nuclei for each culture, for every BPA dose and time condition, and for control cultures. We showed in the present study that BPA disrupted the progression of meiotic prophase in the cultures analyzed, for the two doses at the three time points.

**Figure 6:**
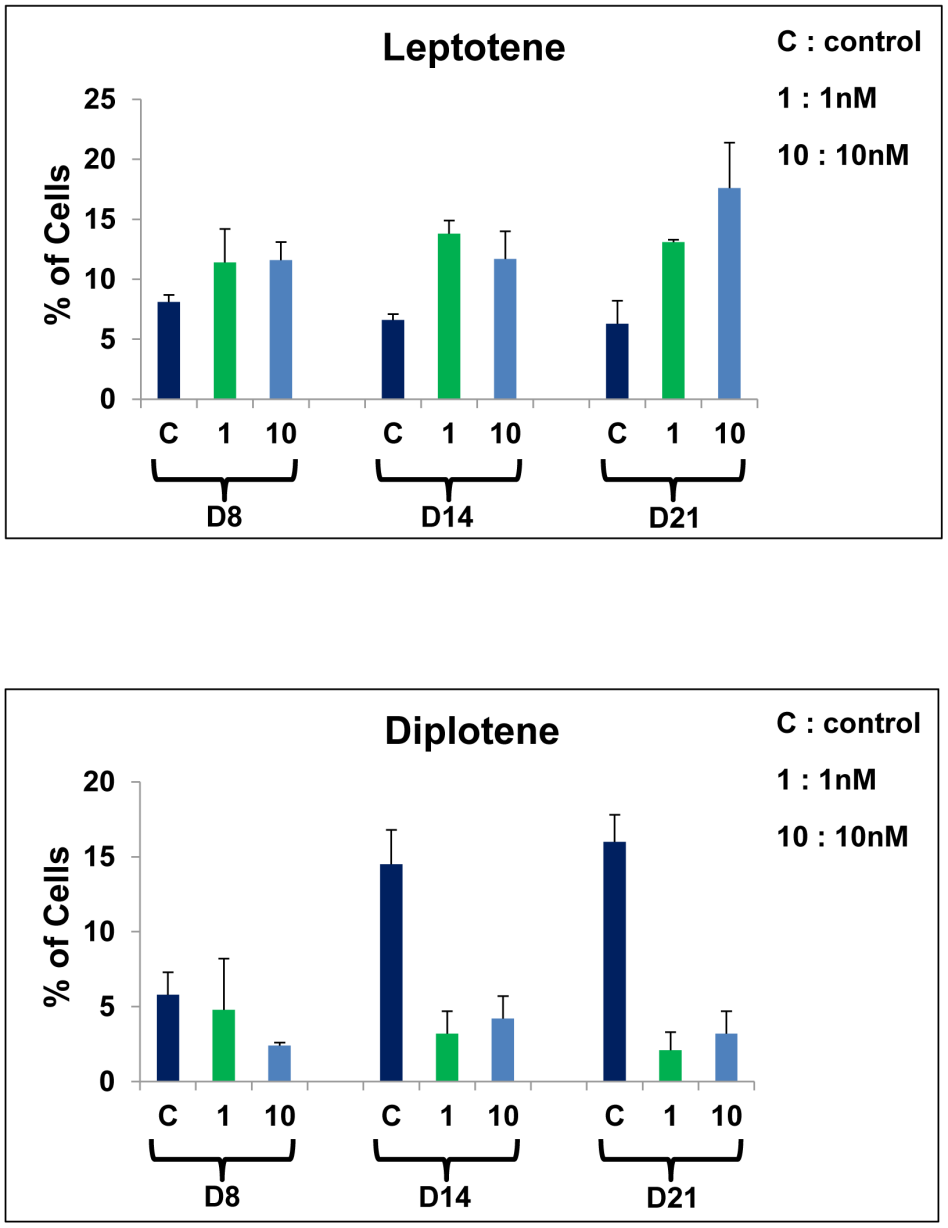
Effects of BPA on the percentages of leptotene and diplotene stages. **A**– percentage of leptotene stage increased at all doses and time points. The increase was at the limit of significance for 1 nM at D8 (p = 0.06) and significant for D14 and D21 at the two doses (p<0.05). **B** – diplotene stage percentage decreased at all doses and time points. The increase was not significant for 1 nM at D8 and was significant for D14 and D21 at the two doses (p<0.05). Each bar represents the mean ±SD (n = 3 cultures for D8 and D14, and 2 cultures for D21) versus control. C =  control culture, 1 and 10 = 1 nM and 10 nM BPA, respectively.

The most obvious changes were observed at the leptotene and diplotene stages. In the treated cultures, the percentage of leptotene stage (Fig. 6A) increased for all days and concentrations compared with control cultures. This increase was at the limit of significance for 1 nM at D8 (11.4±2.8 versus 8.1±0.6, p = 0.06). The increase in leptotene stage was significant (p<0.05) for 10 nM at D8 (11.6±1.5 versus 8.1±0.6 in control), for 1 and 10 nM at D14 and D21 (D14: 13.8±1.1 and 11.7±2.3, respectively, versus 6.6±0.5 in control; D21: 13.1±0.2 and 17.6±3.8, respectively, versus 6.3±1.9 in control). In the same cultures, diplotene stage decreased for all days and concentrations compared with control cultures (Fig. 6B). The decrease in diplotene stage was not significant for 1 nM at D8. This decrease was significant (p<0.05) for 10 nM at D8 (2.4±0.2 versus 5.8±1.5 in control), for 1 and 10 nM at D14 and D21 (D14: 3.2±1.5 and 4.2±1.5, respectively, versus 14.5±2.3 in control; D21: 2.1±1.2 and 3.2±1.5 versus 16.0±1.8 in control). Nevertheless, these changes of leptotene and diplotene stages were independent of the BPA concentration and of the exposure time.

Zygotene stage slightly decreased in the BPA-treated cultures, whereas pachytene stage slightly increased, but these variations were not significant, whatever the doses and time points.

#### Effect of BPA on the pachytene index

**Figure 7:**
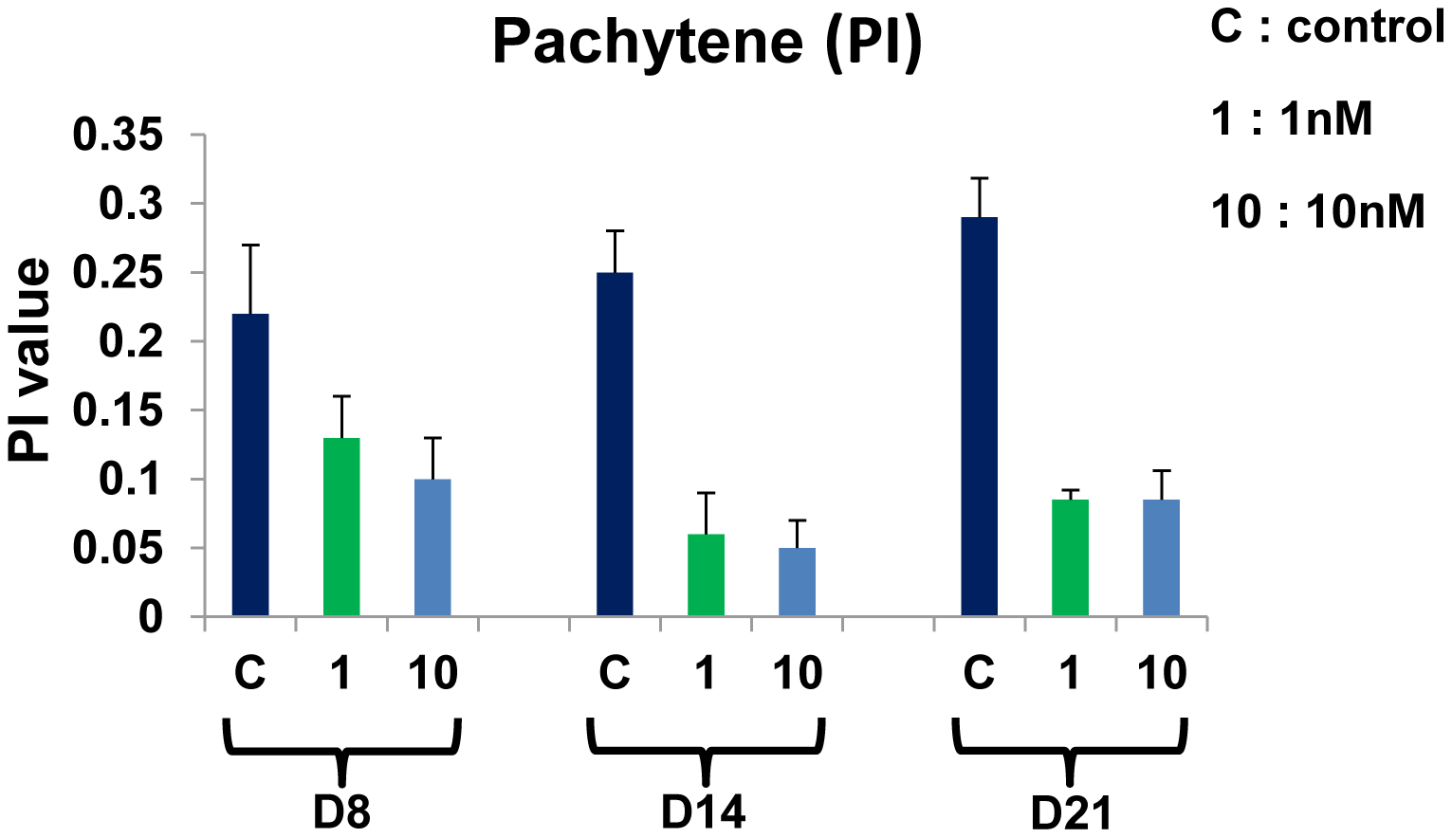
Effect of BPA on the pachytene stage progression. The pachytene index (PI) decreased at both dose and time points (p<0.05). C =  control culture, 1 and 10 = 1 nM and 10 nM BPA, respectively. Each bar represents the mean ±SD (n = 3 cultures for D8 and D14, and 2 cultures for D21) versus control.

Although the percentage of pachytene stage did not vary significantly in this study for the majority of doses and time points, we observed a decrease of the P3 substage at all dose and time points (p<0.05). For 1 nM and 10 nM, at D8: 13.0±2.6 and 10.4±2.3, respectively, versus 22.0±2.6 in control cultures; at D14: 9.3±3.1 and 7.3±2.3, respectively, versus 24.7±3.1 in control cultures; at D21: 8.3±0.2 and 8.3±1.7, respectively, versus 29.1±2.3 in control cultures. Consequently, the PI also decreased at all doses and time points (p<0.05) (Fig. 7). For 1 nM and 10 nM, at D8: 0.13±0.03 and 0.10±0.03, respectively, versus 0.22±0.05 in control cultures; at D14: 0.06±0.03 and 0.05±0.02, respectively, versus 0.25±0.03 in control cultures; at D21: 0.08±0.007 and 0.08±0.02, respectively, versus 0.29±0.03 in control cultures.

#### BPA-induced axial element and SC abnormalities

SYCP3 revealed BPA-induced abnormalities of axial elements and SC at the leptotene, pachytene and diplotene stages.

**Figure 8:**
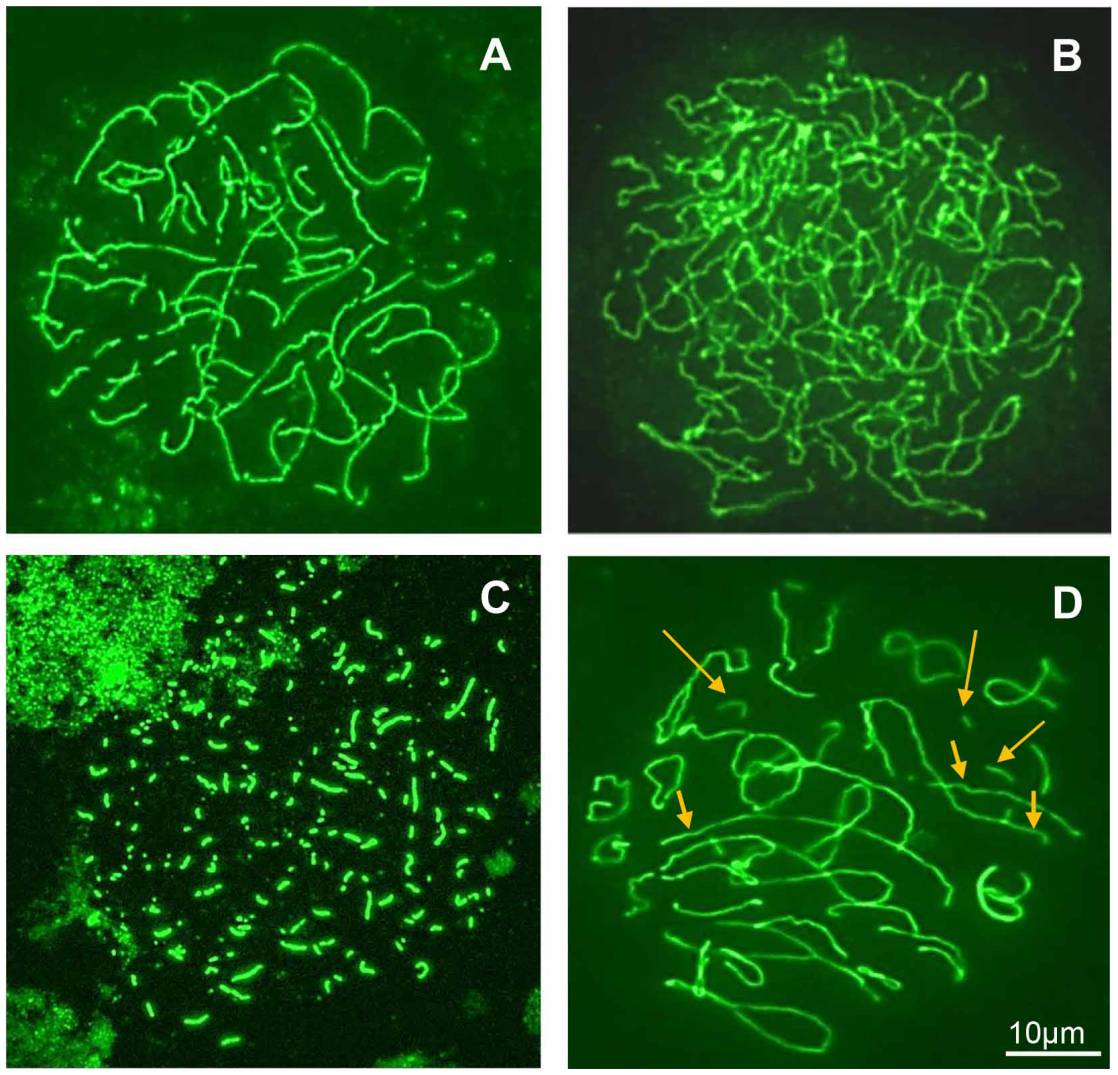
Pictures of abnormal spermatocyte nuclei cultured in the presence of BPA and stained with the anti-SCP3 antibody. **A**– leptotene nucleus with abnormally long stretches of axial cores without indication of polarization; **B** – pachytene nucleus with total asynapsis; **C** – pachytene nucleus with pulverized synaptonemal complexes, proving apoptosis; **D** – diplotene nucleus with univalents (short arrows) and fragments (long arrows). Bar = 10 µm.

**Figure 9:**
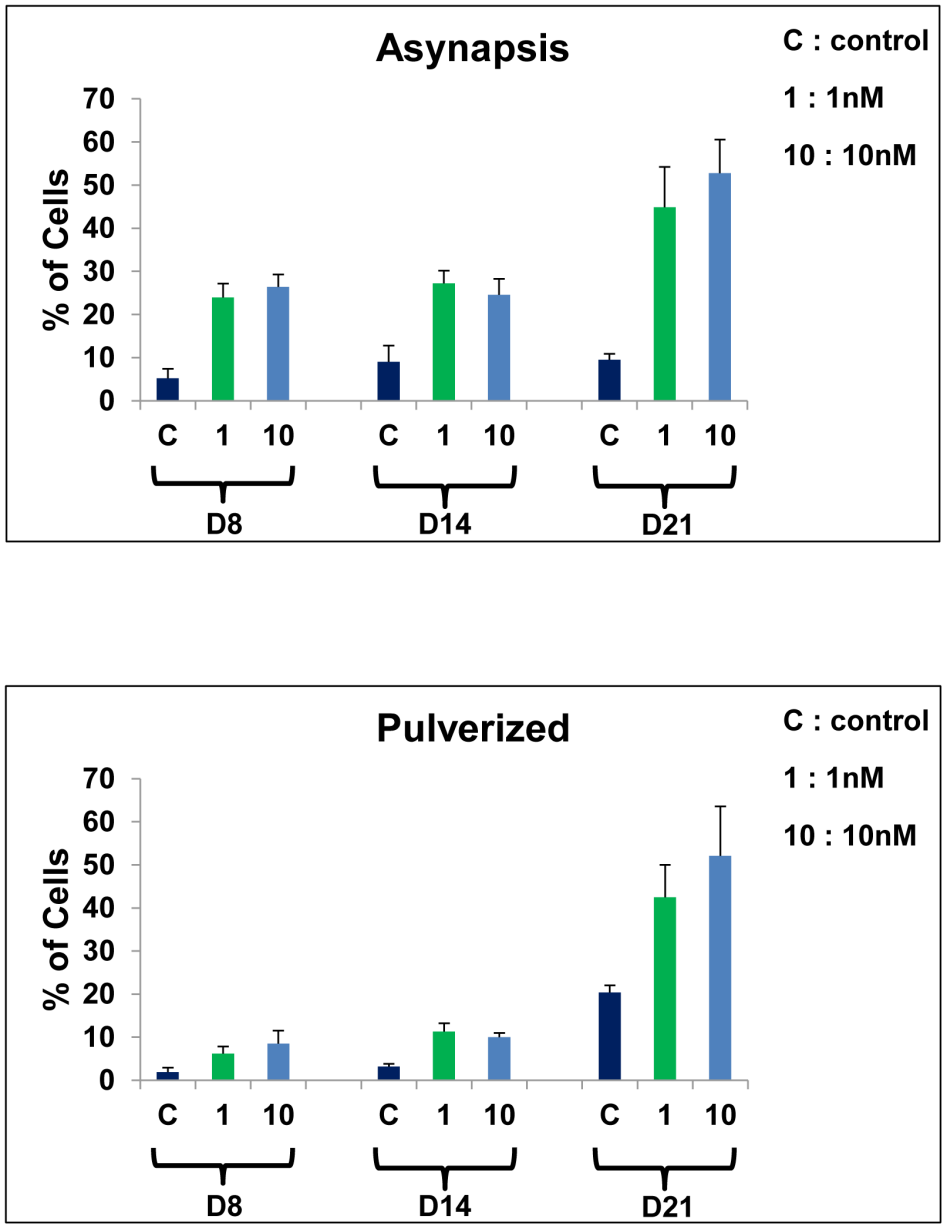
Changes of synaptonemal complex (SC) abnormality percentages during the pachytene stage in BPA-cultured spermatocytes. **A**– the percentage of nuclei with asynapsed SC increases at each dose and time point (p<0.05). **B** – the percentage of nuclei with pulverized SC (apoptotic nuclei) increases at each dose and time point (p<0.05). Each bar represents the mean ±SD (n = 3 cultures for D8 and D14, and 2 cultures for D21) versus control. **C** =  control culture, 1 and 10 = 1 nM and 10 nM BPA, respectively.

At *leptotene*, in the absence of BPA, thin and discontinuous axial cores held the nucleus area of the leptotene nuclei [36], [37]. With BPA, abnormally long stretches of axial cores without indication of polarization appeared in these nuclei at both BPA concentrations and at three culture time points (Fig. 8A). For 1 nM and 10 nM at D8: 25.9±1.2 and 30.2±1.3, respectively; at D14: 22.4±2.1 and 34.9±2.1, respectively; at D21: 23.0±6.7 and 26.5±6.1, respectively.
*At pachytene*, the prevalent abnormality observed in the presence of BPA was asynapsis, especially total asynapsis (Fig. 8B). The percentage of asynapsis increased significantly (p<0.05) for all doses and time points with no dose or time dependency. For 1 and 10 nM, at D8: 24.0±3.2 and 26.4±2.8, respectively, versus 5.2±2.2 in control cultures. At D14: 27.2±2.9 and 24.6±3.7, respectively, versus 9.1±3.7 in control cultures. At D21: 44.8±9.4 and 52.8±7.8, respectively, versus 9.5±1.4 in control cultures (Fig. 9A).
The pulverized SC nuclei (Fig. 8C), proving apoptosis [49], significantly increased (p<0.05) for all doses and time points (Fig. 9B). For 1 nM and 10 nM at D8: 6.2±0.8 and 8.5±3.0, respectively, versus 1.9±0.5 in control cultures; D14: 11.3±1.9 and 10.0±1.0, respectively, versus 3.2±0.6, in control cultures; D21: 42.5±7.5 and 52.1±11.5 versus 20.4±1.6 for 10 nM).
*At diplotene*, all spermatocytes contained univalents and fragmented lateral elements of SC (Fig. 8D).

## Discussion

BPA effects were investigated on male meiosis, using a validated and reproducible seminiferous tubule culture model [35]. Under the present experimental ex-vivo conditions, testosterone, produced in vivo by the Leydig cells and acting on the Sertoli cells, is added directly to the culture medium as described in Staub et al. (2002). As for the relationship between the Sertoli cells and the germ cells, we have shown previously that, under our experimental conditions, cellular junctions between the Sertoli cells and the germ cells, which are most important for germ cell differentiation, are maintained [50], [51]. This model allows the analysis of induced responses by germ cell exposure to low doses of toxic substances for three weeks. This period of time corresponds, in the rat, to the development of spermatogenesis and mimics puberty, a critical period of life sensitive to endocrine disruptors. Indeed, a tiny concentration of endocrine disruptors can produce long-term adverse effects on the reproductive system [52]. Seminiferous tubules from 23-day-old Sprague-Dawley rats were used. At this age there are no round spermatids in the rat testes. Thus, we are sure that the round spermatids originate from the meiotic divisions which occurred in vitro [35]. Moreover, we previously showed similarities between the meiotic processes in vivo and ex vivo [36], [37]. We performed transcriptomic analyses in BPA-treated cultures versus controls without any a priori concerning the results, varying the doses and time points. We performed germ cell ICC analyses on the same cultures, at the same doses and time points. We show that the transcriptomic results and morphological observations are consistent. The percentages of the four populations of spermatocytes under the control conditions, as well as the pachytene substages, were found in the present study to be very similar to those described in our previous publications [36], [37]. BPA concentrations were selected on the basis of the concentrations found in human and rat sera, i.e. 0.2 to 20 ng/mL, meaning 1 to 100 nM [1], [2], [3], [4], [6], [33], [53].

### BPA alters important biological functions and canonical pathways in the seminiferous tubule cultures

The overall number of altered genes is a very good indicator of the level of cellular disturbance induced by a toxic compound [43], [54], [55]. Here, we observed no dose dependency in terms of number of significantly differentially expressed genes, but a time dependency (Fig. 1).

Radar plots showed that BPA alters important biological functions. We analyzed, for all doses and time points, the distribution of altered genes per function, as defined in Gene Ontology. The same functions were altered at each dose and time point, but the number of genes increased only with the exposure time (Fig. 2). Altered genes involved in cancer, cell death and cellular development were predominant. Notably, genes involved in disease of the reproductive system and in DNA replication and repair were also altered.

Although the functions described provide valuable information on the action of the involved genes, the canonical pathways help in understanding, in a faster and more drastic manner, the interactions between these genes themselves and the cellular mechanisms to which they belong. As shown in Fig. 3, the nine main disturbed canonical pathways in our cultures support the literature findings. The disturbance of these canonical pathways was time dependent. Apart from these observations, analyzing the direction of expression (induction or repression) of each specific transcript is difficult in the context of current knowledge. Since the mRNAs are produced in an oscillatory manner (in bursts) [56], their production is likely to obey extremely finely tuned processes, depending on many features, and should not be overinterpreted at this stage.

The *aryl hydrocarbon receptor signaling* pathway was altered as expected, as BPA is an aromatic substance. The *xenobiotic metabolism signaling* pathway by cytochrome P450 was highlighted, as expected, including P450-family genes. Tumor necrosis factor, Tnf, was strongly increased, up to 39 times at D14/1 nM BPA. Several genes of the Nfkb family were upregulated at all doses and time points. Seventy genes encoding heat-shock proteins were mobilized. Their role is to prevent protein aggregation under stress conditions.

The strong activation of *the NRF2-mediated oxidative stress response* pathway induced the overexpression of genes encoding Phase I and II metabolizing enzymes, as well as antioxidant proteins.

The *LXR/RXR activation* pathway was highly disturbed, meaning an effect of BPA on lipid metabolism, inflammation and cholesterol metabolism through the low-density lipoprotein receptor (LdlR). Genes regulated by LXR included ATP-binding cassette transporter A1 (Abca1), which was upregulated. This observation confirms previous descriptions of the implication of BPA in lipid metabolism [57], [58].

An interesting finding was the alteration of the *Androgen signaling* pathway as proof of the role of BPA as an endocrine disruptor. At D21/1 nM BPA, the androgen receptor (AR) was downregulated (−2.7), and the androgen signaling pathway included 76 altered genes. BPA has also been described as an estrogen-like substance [9], [11]. Indeed, in our hands, BPA leaves the marks of endocrine disruption. Numerous prolactin family genes were repressed, especially at 21 days of culture and more intensely at 1 nM than 10 nM. BPA altered the expression of tens of GPCR. At D21/1 nM BPA, the estrogen receptors, Esr1 and Esr2, were also significantly downregulated (−15.3 and −14.3, respectively). Members of the aldo-keto reductase family (Akr1c), and especially Akr1c12/C13 and Akr1c3, were downregulated. This late gene encodes an enzyme essential for testosterone synthesis and could explain the decreased testosterone synthesis already described with BPA *in vivo* [52].

One can, logically, wonder why these features are visible at 1 nM and not at 10 nM. A lack of dose dependency is often a mark of endocrine disruptors, which induce nonmonotonic dose-response curves [5]. An endocrine disruptor can be equally as potent as endogenous hormones in some systems, causing biological effects at levels as low as picomolar [59]. One of the possible explanations for the absence of dose dependency might be that, after the receptor is bound by BPA and transcription of target genes has occurred, the reaction eventually must reach a plateau until the bound receptor must be inactivated [60]. As an antiandrogen, BPA can also trigger other mechanisms of action that are more complex or even antagonist to the previous one [61].

Many genes were implicated in genetic recombination, in particular the process of *DNA double-strand break repair by homologous recombination*, and are discussed below. The *role of BRCA1 in DNA damage response* is also a major altered pathway and is consistent with the many genes playing a role in cancer functions. Atm, Atr and Chk2, regulators of the DNA damage response, were downregulated in several experimental conditions (Table S1). Thus, our transcriptomic analysis on cultured seminiferous tubules supports current knowledge regarding BPA action.

### Transcriptomic analysis sustains immunocytological observations

We showed in this study that ICC analysis revealed concomitant modifications of meiotic prophase chronology, defects of chromosome pairing, and induction of germ-cell apoptosis in cultures exposed to BPA. Combined transcriptomic analysis showed that BPA deregulates numerous genes involved in premeiotic steps and in the first meiotic prophase: DNA replication and sister chromatid cohesion, initiation of meiosis, pairing and genetic recombination, meiotic control and checkpoints, germ-cell apoptosis, and genes implicated in several other cellular processes essential to the life of the germ cells (Fig. 4). In the scope of this article, to explain the abnormalities revealed by ICC, we chose to focus in particular on 120 genes, preceding meiotic divisions, involved in the first meiotic prophase, or essential to meiosis (Table S2). This table shows that differentially expressed genes are almost the same at both doses of BPA for a same time of exposure. This observation and the fact that most of these genes/proteins interact with each other as shown in Figure 5 could explain why the phenotype observed by ICC is the same for 1 and 10 nM.

Quantitative analysis of meiotic prophase revealed that BPA increases the percentage of leptotene nuclei, one-third of these showing abnormally long stretches of axial core. Abnormalities of the leptotene stage could be consistent with an alteration of cells to progress towards the zygotene stage. According to Zickler and Kleckner [62], the leptotene/zygotene transition appears to be an unusually complex and critical transition. Indeed, during this transient period the polarization of meiotic chromosome telomeres (bouquet stage) occurs, which is essentially concomitant with the onset of synaptonemal complex (SC) formation. This stage is also concomitant with the progression between DSBs and stable strand-exchange intermediates (double Holliday junctions). These observations are consistent with the microarray analysis, showing transcriptional changes of the genes involved in the early steps of genetic recombination as a homology search, DNA strand exchange and stable Holliday junction formation (Fig. 4). Thus, it is impossible for most germ cells to pass through the leptotene-zygotene transition. The most deregulated gene, Stra8 (Table S2), participates as a fundamentally positive regulator in the commitment of spermatocytes to meiosis, and regulates progression through the early stages of meiotic prophase [63]. In the study of Mark et al. [63], a small percentage of *Stra8*-/- spermatocytes progressed into the later stages of meiosis and showed a prolonged bouquet stage configuration. Leptotene nuclei with abnormally long stretches of axial core were also reported in Dmc1-/- mice [64]. In our study, Dmc1 was strongly repressed at D21/1 nM BPA and upregulated at D21/10 nM. The regulation of some key genes of meiosis was validated by qRT-PCR (Table 1).

Morphological analysis of pachytene spermatocytes indicated the prevalence of asynapsis – almost exclusively extended asynapsis. Homologous pairing failure has also been described in pachytene oocytes of C.elegans and mice exposed to BPA [25], [30], [65]. This abnormality is obviously related to the changes we observed in the transcription of many genes involved in homologous pairing and recombination. We previously reported [66] that high levels of extended asynapsis could arise from defects affecting these two crucial steps of meiosis. In the particular case of BPA, Allard and Colaiacovo [65] and Liu et al. [31] postulated that the DSB's repair machinery is impaired. In the present study, we showed that several genes involved in this process were deregulated by BPA (Table S2, Fig. 4). Several members of the Rec/Rad-51 family, which is known to play an important role in DNA repair by homologous recombination, were up- or downregulated, with a high fold change for Rad51C. Genes involved in the DNA damage response, such as Brca1, Atm, Atr and Chk2, were deregulated. Nevertheless, other impaired processes may be involved in asynapsis formation. We showed that several genes involved in DSB generation (Spo11), in homology search and DNA strand exchange, and in the formation of Holliday junctions (Exo1, Hormad2, Msh4, Rad51C, Rad52, Tex15) were strongly deregulated. The gene, Fpr3, which functions in a checkpoint-like manner to ensure that chromosome synapsis is contingent on the initiation of recombination, was strongly underexpressed. Of the proteins of genetic recombination, the SC proteins can be directly involved in the mechanisms of asynapsis. Indeed, the corresponding genes, Sycp1, Sycp2, Sycp2L and Sycp3, were clearly downregulated. It has also been reported that Stra8 plays a direct role in SC assembly [63]. The above discussion about asynapsed pachytene could explain the presence of univalents at the diplotene stage. Deregulation of genes which stabilize the Holliday junction (Msh4, Msh5) or maintain genomic integrity during DNA replication and recombination (Mlh1, Mlh3) could be involved in diplotene abnormalities. We emphasize that all these genes interact with each other. Their interconnections were visualized in a literature-based network showing the high level of connections between differentially expressed genes linked to DNA DSB repair in our datasets (Fig. 5). Thus, the phenotypes observed with ICC could result from the action of several of these interconnected genes and probably other genes expressed in germ cells and whose functions are not yet clearly defined.

Expression changes of genes revealed in our study could also explain some published data. We saw above that the increased number of Rad51 foci observed by Allard and Colaiacovo [65] might be consistent with transcriptional changes of the Rec/Rad51 family. The increased number and modified distribution of Mlh1 foci reported in pachytene oocytes following BPA exposure [25], [30] might be consistent with the strong downregulation of Prdm9, a major player in hotspot specification [67].

Morphological analysis revealed the presence of apoptotic (pulverized) spermatocytes (Fig. 8C) whose percentage significantly increased at D21. Early features of apoptosis were also observed with the transcriptomic analysis. Radar plots (Fig. 2) show that cell death is one of the most represented functions within the deregulated genes. Among the BPA-responsive apoptosis genes, we identified genes from the Card and Casp families. The antiapoptotic gene, Bcl2, was downregulated whereas the proapoptotic gene, Bax, was upregulated (Table S2). In addition Nfκb, which modulates the differentially expressed Card-family genes, was strongly induced.

Apoptosis might be induced in cells that fail to recombine and/or pair their homologous chromosomes [68]. Several checkpoints that monitor the progression of meiotic recombination are activated in response to the unrepaired meiotic DSBs. For example, Atm and Atr are checkpoint kinases that regulate meiotic DSB repair; both were downregulated in our study. Thus, the accumulation of apoptotic spermatocytes coupled with a decrease of the pachytene index is an indication of pachytene checkpoint activation by BPA. We have localized this checkpoint at the end of the P2 pachytene substage in humans [66] and rats [37]. The majority of asynapsed spermatocytes could, thus, be eliminated in this way. This could explain the decreasing percentage of diplotene nuclei in our BPA-exposed cultures.

These results demonstrate the interest of the analysis of SC in toxicological studies because they underline the specificities of each toxic substance. SC analysis is a highly sensitive indicator of potentially heritable effects of genotoxic agents [69]. All agents tested by Allen et al. [70], [71] and Backer et al. [69] caused dose-dependent SC damage, which varied with the chemicals. We also previously showed with the same culture model that Cr (VI) treatment led to SC fragmentation whereas Cd treatment induced moth-eaten SC [36], [37]. Presently, we show that BPA alters meiotic cell progression and increases asynapsis, without dose dependency.

### Does BPA produce aneuploid gametes?

The pachytene checkpoint prevents chromosome missegregation by eliminating asynapsed pachytene spermatocytes that would lead to the production of aneuploid gametes [68]. Nevertheless, as shown in Fig. 4, BPA elicited expression changes of 11 genes implicated in pachytene checkpoint function, leading to a failure of checkpoint activation that could alleviate the meiotic arrest at this point.

Moreover, we previously showed that the pachytene checkpoint was not an absolute barrier [66], abnormal meiotic cells being able to complete spermatogenesis. In the present study, the presence of diplotene spermatocytes with univalents leads to the assumption that the existence of aneuploid metaphases II cannot be excluded. If so, low-level BPA exposure could induce errors in chromosome segregation and could produce aneuploid germ cells. Consistent with this hypothesis, studies of oocyte meiosis from female mice exposed to BPA indicate that BPA can affect chromosome segregation by disturbing synapsis and recombination [28], [30]. A second mechanism involving the cell division machinery was suspected to explain a potential aneugenic effect of BPA [26]. It was demonstrated that BPA alters the centrosome dynamic and increases the number of mitotic and meiotic spindles with unaligned chromosomes. Nevertheless, according to Eichenlaub-Ritter et al. [27], [72], low-level chronic BPA exposure does not appear to pose a risk for the induction of errors in chromosome segregation at first meiosis in mouse oocytes. These authors preferentially suggested that BPA induced meiotic arrest. However, we did not find in the literature any sperm chromosomal analyses of BPA-exposed male rodents to demonstrate the existence of aneuploidy.

According to Hunt et al. [73], studies in rodents allow predictions about humans to be made regarding reproductive effects of EDCs. Chalmel et al. [74] reported a cross-species expression profile between rodents and humans. Thus, our cytological and transcriptional results could be a predictor of the deleterious effects of low-dose BPA on human spermatogenesis. Another point is that, although *ex vivo* models might be questionable for their lack of biotransformation and clearance compared to *in vivo* models, they do nevertheless represent a good alternative to animal testing regarding the necessary reproductive toxicity assays of thousands of chemicals.

## Conclusion

This study provides arguments for the deleterious effects of BPA at low doses on male germ cells, by combining transcriptomic analyses and immunocytochemistry in an *ex vivo* rat seminiferous tubule culture model. Transcriptomic analyses showed that BPA altered the expression of genes involved in events preceding meiosis, its initiation and progression. Of the numerous genes differentially expressed by low-dose BPA exposure, we focused on 120 premeiotic and meiotic genes; some showed very elevated fold changes. Nevertheless we did not observe any dose dependency between 1 nM and 10 nM with both the techniques used. Only the gene expression analysis underlined a time dependency between D8, D14 and D21. Immunocytochemistry showed that low-dose BPA had deleterious effects on meiotic progression, and that the main alterations induced by BPA are asynapsis and apoptosis. These results bring additional arguments for the hypothesis that BPA alters pairing and recombination. Moreover, many differentially expressed genes were also involved in other important physiological functions, corroborating published findings, such as the triggering of xenobiotic metabolism, the disturbance of lipid metabolism, and endocrine disruption. Further analysis of our transcriptomic data could help to provide candidates for predictive biomarkers of meiotic abnormalities related to the toxic effects of chemicals on spermatogenesis.

## Supporting Information

Table S1 **List of genes up- or down-regulated at 1 nM and 10 nM BPA (D8, D14 and D21).**
(XLS)

Table S2 **List of the 120 genes differentially expressed under BPA exposure involved in the premeiotic and first meiotic prophase.** Fold change values and cellular localization (N nuclear, C cytoplasm, PM plasma membrane, Un undertermined), are reported for the two BPA concentrations (1 and 10 nM) and at the three time-points (D8, D14 and D21). Red =  up-regulated genes; green =  down-regulated genes. The number of deregulated genes is time dependent but not dose-dependent.
(PDF)
