# Supplementary material for: Exposure to Low-Dose Bisphenol A Impairs Meiosis in the Rat Seminiferous Tubule Culture Model: A Physiotoxicogenomic Approach
Source: PLoS One. 2014 Sep 2;9(9):e106245. doi: 10.1371/journal.pone.0106245 (PMC4152015; doi:10.1371/journal.pone.0106245)
Supplement: Table S2 — List of the 120 genes differentially expressed under BPA exposure involved in the premeiotic and first meiotic prophase. Fold change values and cellular localization (N nuclear, C cytoplasm, PM plasma membrane, Un undertermined), are reported for the two BPA concentrations (1 and 10 nM) and at the three time-points (D8, D14 and D21). Red = up-regulated genes; green = down-regulated genes. The number of deregulated genes is time dependent but not dose-dependent. (PDF) [file pone.0106245.s002.pdf]

Table S2

Scale from -40 to +30

	BPA 1nM			BPA 10nM			
**Genes**	**D8**	**D14**	**D21**	**D8**	**D14**	**D21**	**Localization**
Asz1			-22,05			-4,437	N
Atm	-2,388	-3,24	-1,772				N
Atr				-2,021	2,493		N
Atrx	-1,622						N
Aurkb		-2,834	-6,4	-1,602		-2,512	N
Bax			2,577			1,636	N
Bcl2		-1,614	-2,307			-1,725	C
Bcl2A1		4,908		1,781		-3,689	C
Bcl13		1,625	-8,781	1,76		-2,958	C
Bcl14			-13,446			-3,183	C
Boll			-13,966			-3,939	C
Brca1			-5,905			-2,477	N
Brca2		-2,116	1,957			2,412	N
Btrc			3,443	-1,841		6,75	C
Card6			-3,598	-1,608		-2,408	C
Card9			-3,665	-1,545			C
Card10			-7,786	-2,779	1,611		C
Card11			-16,657			-3,85	C
Casp1		2,641	-4,178	1,62	-1,64	-3,369	C
Casp2		1,928	6,934			-2,899	C
Casp3		2,007	-1,833				C
Casp4	1,606	3,591	5,328				C
Casp6		-1,913	5,153			6,22	C
Casp7			4,331	1,553		4,211	C
Casp8			-3,509			-2,846	N
Casp9			-2,179	-2,096		-1,984	C
Casp14						-2,876	C
Ccna1		-3,365		-1,929			N
Ccna2		-2,972	-5,445			-2,198	N
Ccne2			-9,963	-1,705		-3,294	N
Cdk2						1,5	N
Chk1					-1,709	-2,264	N
Chk2						-3,306	N
Cyp26B1	1,582	2,11	6,67	2,651		8,361	C
Daz2		2,561	-4,001			-2,604	C
Dazap1			2,496			1,634	C
Dazap2		-1,765	2,353	-1,661		2,556	N
Dmc1			-14,059			3,925	N
Ewsr1			4,158			3,009	N
Exo1		-1,776	-14,584			-4,247	N
Fancd2				-1,707			N
Figla		-12,255	-6,375			-2,611	N
Fkbp6			-2,629			-1,927	N
Fpr3			-25,148			-2,595	PM
Gen1	-1,576	-2,052	-1,622				Un
Hist1H2Ba	-1,707	2,982	-8,527	-1,893			N
Hist1H2Bb		3,097	2,195	1,701		3,175	N
Hormad1		1,592	-3,273				N

Table S2

Scale from -40 to +30

	BPA 1nM			BPA 10nM			
**Genes**	**D8**	**D14**	**D21**	**D8**	**D14**	**D21**	**Localization**
Hormad2		1,894	-18,764	-1,632		-5,154	N
Hspa2			-3,246				C
Hus1			-6,816			-3,572	N
Mael			-1,649				C
Mapk14			2,785			3,388	C
Mei1		-2,849	3,02	-1,562		2,963	Un
Mei4			-9,725	-1,686		-3,165	N
Mlh1			-3,108			-2,331	N
Mlh3			4,506			3,977	N
Mre11					1,62		Un
Msh2	1,561	2,028	6,533	2,193			N
Msh3						1,984	N
Msh4	-1,832		-8,396	-1,608		-3,018	N
Msh5			3,017			3,838	N
Nos2	5,192	29,976					C
PapolA		-2,35	2,505	1,503		2,598	N
PapolB	-1,506			-2,204			N
PapolG			2,866	-1,562		2,115	N
Pds5A		1,72		-1,91			N
Pds5B			1,896			2,526	N
Pms1						2,051	N
Prdm9			-25,36				N
Pola1	-2,097		-6,157			3,079	N
Ppp1Cc			3,197			2,537	C
Rad1			2,914				N
Rad21			2,528			2,541	N
Rad21L			-9,433			-3,735	N
Rad51	-1,571	-2,091	-7,496	-1,839			N
Rad51B			-10,651			-2,944	N
Rad51C			-23,733			-3,589	N
Rad52			7,898			5,07	N
Rad54B		-1,721	-7,034			-2,844	N
Rec8		-2,856					N
Rpa1	-1,509			-1,819			N
Rpa2			1,552				N
Smc1A			1,857				N
Smc2			-6,353				N
Smc3			3,079			3,334	N
Smc4			-4,962			-1,995	N
Smc5				1,633		4,444	N
Sohlh1	-1,724	-1,706	-2,012	-1,875			C
Spo11			-8,241				N
Stag3			-3,457				N
Stmn1						3,213	C
Stra8	-2,155	-4,215	-37,833	-1,877	1,67	-4,607	Un
Sumo1			2,238			2,521	N
Sumo2		-1,61	2,531	1,519	-1,539		N
Sumo3			3,701			3,109	N

Cont.

Table S2

Scale from -40 to +30

	BPA 1nM			BPA 10nM			
**Genes**	**D8**	**D14**	**D21**	**D8**	**D14**	**D21**	**Localization**
Syce1	-1,541						N
Syce2		4,13					N
Sycp1	-1,561		-9,13				N
Sycp2	-1,619	-2,458	-5,862				N
Sycp2L			-14,877			-3,415	Un
Sycp3		2,091	-4,717				N
Syn1			-10,866			-3,606	PM
Terc		2,512	-7,72		-1,772	-3,053	Un
Tex11		-1,728	-11,193	-1,724			N
Tex12			-11,082	-1,727		-3,43	N
Tex 13B		-1,526	-10,23	-1,967		-3,091	C
Tex14			-10,377	-1,548		-2,928	PM
Tex15		-4,065	-20,549			-3,134	Un
Tex19	-1,644	2,169	4,005	-1,586		4,181	N
Tex101			-5,008			-2,03	PM
Top2A			-4,829				N
Top2B			-6,763			-2,941	N
Top3A			-3,1		2,026		N
Top3B			5,67			6,133	N
Trip13	-1,526						C
Ube2B			2,98			2,932	C
Ubr2	1,65		4,523	1,503		4,068	Un
Wee1		-1,518	4,159		3,14		N
Wee2			-8,882		-2,474	-3,276	C
